# Effect of commercially pure titanium implant coated with calcium carbonate and nanohydroxyapatite mixture on osseointegration

**DOI:** 10.25122/jml-2022-0049

**Published:** 2023-01

**Authors:** Zainab Saleh Abdullah, Mustafa Shaker Mahmood, Faiza Mohammed Ali Abdul-Ameer, Abdalbseet Ahmad Fatalla

**Affiliations:** 1 Department of Prosthodontics, College of Dentistry, University of Baghdad, Baghdad, Iraq

**Keywords:** calcium carbonate, electrophoretic, osseointegration, nanohydroxyapatite, histomorphometric

## Abstract

In this research, rabbit femurs were implanted with CP Ti screws coated with a combination of CaCO_3_ and nanohydroxyapatite, and the effect on osseointegration was assessed using histological and histomorphometric examination at 2 and 6 weeks. CaCO_3_ and nanohydroxyapatite were combined with the EPD to coat the surfaces of the CP Ti screws. The femurs of five male rabbits were implanted with coated and uncoated implant screws. Healing time was divided into two groups (2 and 6 weeks). After 2 and 6 weeks of implantation, the histological examination revealed an increase in the growth of bone cells for coated screws, and the histomorphometric analysis revealed an increase in the percentage of new bone formation (after 6 weeks, 5.08% for coated implants and 3.66% for uncoated implants). In addition, the uncoated implant, the CP Ti implant coated with a combination of CaCO_3_ and nanohydroxyapatite, stimulated early bone development after two weeks and mineralization and maturation after six weeks.

## INTRODUCTION

Dental implants are essential therapeutic strategies for restoring the appearance and function of dentition. Osseointegration is a fundamental concept in modern implant dentistry and orthopedics and refers specifically to the connection between ordered, living bone and the surface of a load-bearing artificial implant. This connection is characterized by forming a thin layer of bone tissue around the implant, which becomes firmly attached to the implant over time without fibrous tissue in between [1, 2]. Clinically, osseointegration is considered when an implant makes direct contact with a bone while remaining immobile. The lack of a locally or systemically inflamed reaction histologically indicates osseointegration [3, 4].

In addition to the primary mechanical bonds that act over an osseointegrated implant, the success of osseointegration depends on the formation of strong chemical bonds. These chemical bonds can form as bone tissue grows into the irregularities of the dental implant's surface, which leads to three-dimensional stabilization. A unique topography of implant surfaces has been designed by researchers to enhance osteoblastic migration, adhesion, proliferation, and differentiation. Technologies for enhancing bone growth on the implant surface and accelerating osseointegration have developed quickly in the field of surface dental implants [5, 6].

Failure of osseointegration can result in pocket formation around an implant's coronal region and crestal bone loss. The surface of a dental implant is typically modified to produce a consistent layer composed of several coating materials. Surface changes can be made via mechanical, physical, and electrical techniques [7, 8].

This study used calcium carbonate, a white, odorless chemical, as one of the coating materials. Calcium carbonate is a biocompatible material. The other substance was nanohydroxyapatite, a ceramic biomaterial that is bioactive and brittle. Electrophoretic deposition (EPD) is one of the most widely used coating techniques since it is simple to apply and inexpensive [9, 10].

Bone-related proteins, noncollagenous matrix layers, mesenchymal cell migration, and attachment to implant surfaces all contribute to the control of cell adhesion and mineral binding [11].

The peri-implant tissue was histologically analyzed using a light or transmission electron microscope on decalcified or ground slices (TEM). The quantities of peri-implant bone and bone-implant contact in a dyed specimen of the implant and peri-implant bone were accurately measured by histomorphometric analysis. [12, 13]. The osteoconductive evaluation of an implant under a light microscope is useful when ground sections are used. The percentage of bone-to-implant contact and bone area percentage inside threads were measured using histomorphometric analysis [14].

## MATERIAL AND METHODS

### Implant preparation

Twenty screw-shaped implants were created from titanium bars using a lathe (the threaded part was 2.58 mm in diameter). The screws were divided as follows:

- Control group: uncoated screws [five screws for each interval (2 and 6 weeks)] (n=10);
- Experimental Group: a mixture of CaCO_3_ and nanohydroxyapatite were deposited on the screws through electrophoretic deposition (n=10) (five screws for each interval of 2 and 6 weeks).

A coating of CaCO_3_ and nano-hydroxyapatite was applied to the surfaces of the CP Ti screws using EPD at 60 V for 30 seconds. For the coated screws, densification occurred at 400°C for one hour [15]. An autoclave was utilized for screw sterilization for 35 min at 134°C and 2 bars [16].

### The surgical procedure of implantation

**Figure 1:**
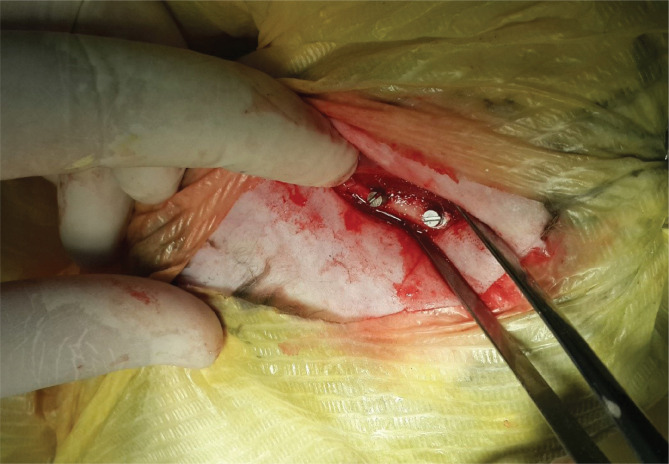
Coated and uncoated implants in position.

Five male rabbits weighing around 2 kg each, with femur bones, received coated and uncoated implant screws. The external surfaces of the femurs were shaved, and a cut was made on the lateral side. The bone was penetrated using a 1.3 mm diameter round bur. One centimeter apart, two holes were created. Coated and uncoated screws were inserted into the upper and lower holes using a screwdriver until fully embedded in the bones (Figure 1).

### Sample preparation for histological examination

An incision was made 0.5 centimeters from the implant screw to obtain the bone-implant block. The blocks were first kept in 10% formalin, followed by daily changes of 8% formic acid until full decalcification. The implant screw was gently withdrawn from the bone bed. The bone was divided across. The specimen was washed for one hour under running water after being placed in a tiny lidded basket. The specimen was dehydrated by setting it in a dish of alcohol that had progressively higher alcohol concentrations (70, 80, 90, and 100%). The samples were heated at 58 and 60°C in an oven for 30 minutes after being exposed to three changes of xylene for one hour. The tissue was then filled with xylene and paraffin wax. The specimens were positioned in the middle of the paraffin blocks. Hematoxylin and eosin were applied to the slide, and it was stained for 10 minutes. The sections were photographed at 4x and 20x their original size under a light microscope.

### Histomorphometric analysis

**Figure 2:**
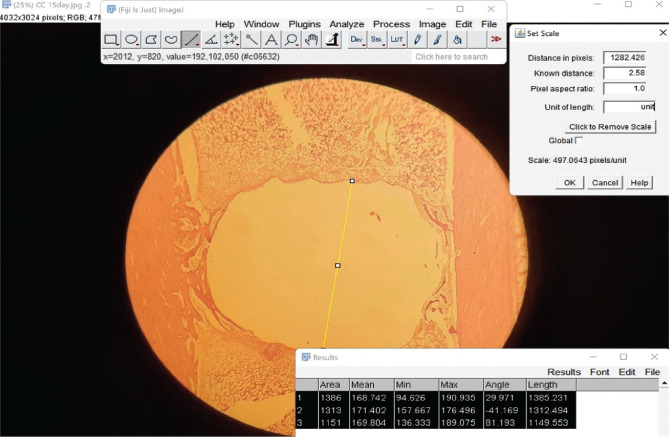
Measuring the section diameter.

**Figure 3:**
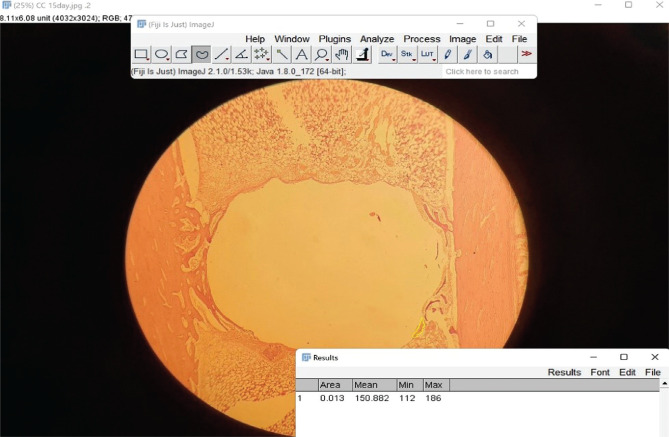
New bone area tracing.


After a six-week healing period, histomorphometric analysis (the assessment of the percentage of new bone growth) was carried out using a Fiji ImageJ program (version 1.53k). First, the diameter of the section was measured, and the mean value was added to the scale box with the screw's diameter (thread part). The values were saved in the software as area measurement data (Figure 2). Then the new bone areas were outlined and measured with a Fiji ImageJ program (Figure 3). The new bone formation percent (NBFP) was calculated using fifty sections from each interval with the formula below [17]:

NBFP%=×100

## RESULTS

### Histological features of implanted areas during different intervals

#### Two weeks after uncoated screw implantation

Low magnifications (H&E, magnification 4x) revealed native bone with the implant interface and early osteoid development at the periphery of the native bone 2 weeks after implantation. The implant site was surrounded by natural bone with local osteoid development, fibrous tissue (spindle cell growth), and scattered capillaries visible at high magnification (20x).

#### Two weeks after coated screw implantation

**Figure 4:**
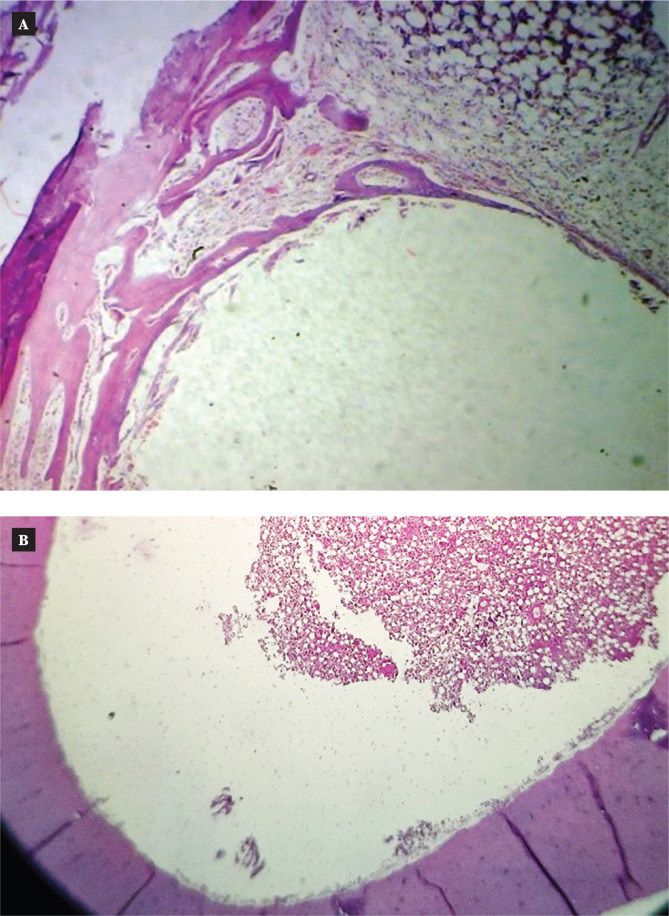
Microscopic view of the uncoated implant in the thread area of rabbit femur two weeks after implantation (H&E stain) 4x. A: Control; B: Coated.

**Figure 5:**
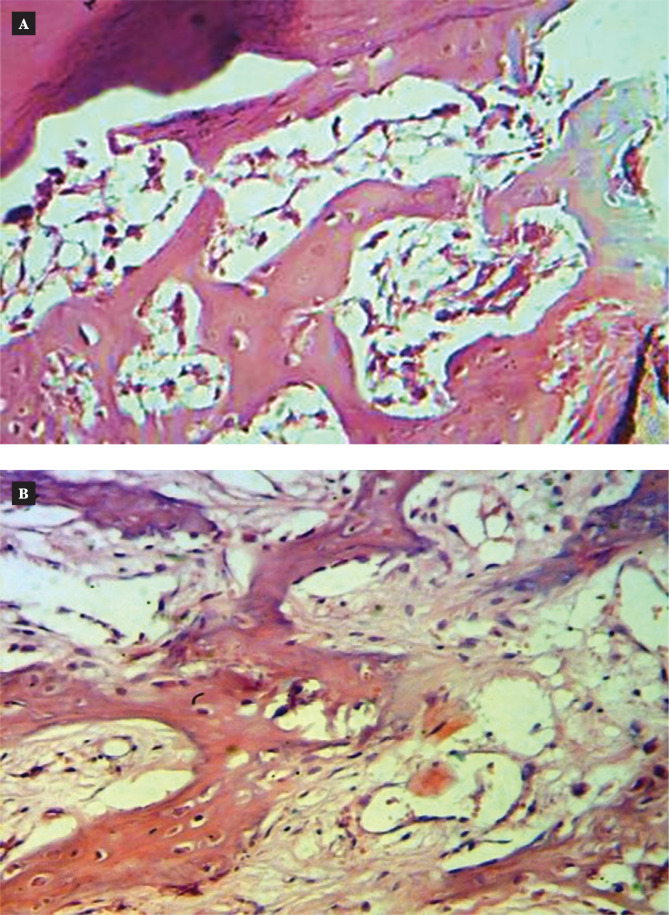
Microscopic view of the uncoated implant in the thread area of rabbit femur two weeks after implantation (H&E stain) 20x. A: Control; B: Coated.

After two weeks of implantation, low magnification (H&E, magnification 4x) of the slice revealed native bone at the implant interface (implant space). The natural bone around the implant site had areas of spindle cell proliferation at the interface and showed a small increase in osteoid tissue when viewed under a high magnification (20x) lens (Figure 4 A, B and Figure 5 A, B).

#### Six weeks after uncoated screw implantation

Low magnification (H&E, magnification 4x) revealed native bone with the implant interface and osteoid development at the native bone boundary six weeks after implantation. The implant space was surrounded by mature bone trabeculae with a significant reversal line and fibroblast at the interface and revealed calcification tissue with conspicuous osteoblast and osteocyte lacunae at high magnification (20x power).

#### Six weeks after coated screw implantation

**Figure 6:**
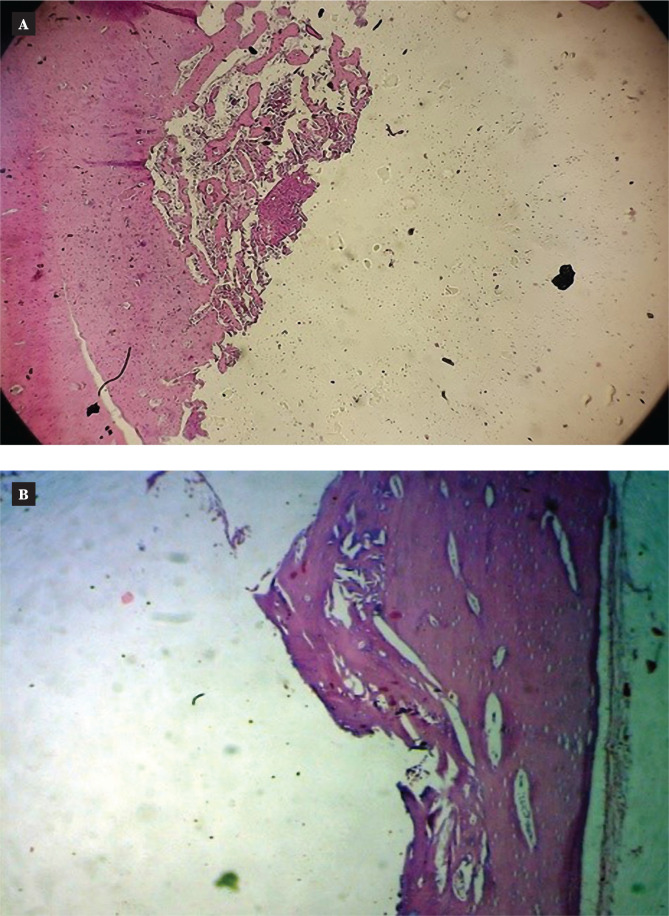
Microscopic view of the uncoated implant in the thread area of rabbit femur six weeks after implantation (H&E stain) 4x. A: Control; B: Coated.

**Figure 7:**
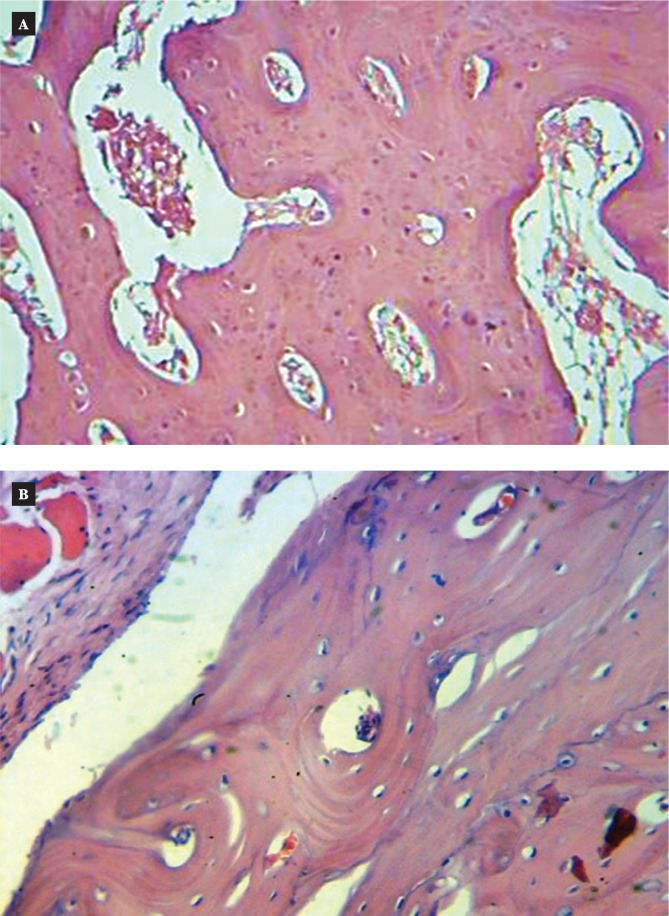
Microscopic view of the uncoated implant in the thread area of rabbit femur six weeks after implantation (H&E stain) 20x. A: Control; B: Coated.

Six weeks after implantation, the section revealed native bone at the implant interface (implant space) at low magnifications (H&E, magnification 4x). The implant space was surrounded by natural bone at high magnification (20x), which also revealed an increase in the osteoid tissues around the implant space and variably thicker spindles of spindle cell proliferation at the interface. There was a noticeable reversal line with areas of calcification, osteoblastic activity, and osteoclastic activity (Figure 6 A, B and Figure 7 A, B).

### Histomorphometric analysis

The NBFP of the CaCO_3_ and nanohydroxyapatite mixture-coated implants in the rabbit bone was greater than that of the uncoated implants 6 weeks after implantation. The mean value of the NBFP of the coated implants was 5.08% *versus* 3.66% for the uncoated implants (Table 1).

**Table 1:** Descriptive analysis of NBFP for CaCO_3_ and nano hydroxyapatite-coated and uncoated groups after six weeks.

Types	No.	Mean	S.D.	S.E.	Min.	Max.
**Uncoated**	5	3.668	±0.642	0.090	2.498	4.912
**Coated**	5	5.086	±0.807	0.114	3.741	6.321

There was a highly significant difference in the mean values of NBFP between CaCO_3_ and nano-hydroxyapatite mixture-coated and uncoated implants after 6 weeks (Table 2).

**Table 2:** Difference in the mean values of NBFP between CaCO_3_ and nano hydroxyapatite-coated and uncoated implants after six weeks.

Student's t-test	t	df	P-value	Sig. (2-tailed)	Mean Difference
**Coated–uncoated**	9.716	8	0.000	HS	1.417

HS – Highly significant at p≤0.001.

## DISCUSSION

The popularity of CP Ti implants is given by their desirable physiochemical and biocompatible characteristics [18]. Furthermore, it is an efficient technique for topographical and chemical surface modification to increase the bioactivity of dental implants [19]. Due to its great biocompatibility, excellent bio affinities, osteoconductivity, and chemical resemblance to human bone and teeth, the hydroxyapatite coating combination is regarded as a potential biomaterial [20]. Inorganic material, CaCO_3_, and biocompatible nutrients are included in the list of compounds that may be used as dietary supplements for dilatory purposes [21].

The histomorphometric analysis is one of the invasive techniques used to examine implant tissue [22]. It may be utilized at any time to evaluate the stability of an implant [23]. Biomaterial surface features impact the biomaterial interface for cell response impacting the growth and production of new bone [24]. Implant-based tooth replacement is an essential procedure for both cosmetic and practical reasons [25]. Because of its excellent biocompatibility, good bio-affinity, osteoconduction, and crystallographic and chemical resemblance to human bone and teeth, hydroxyapatite has been recognized as a promising biomaterial. However, one of the biggest barriers to more widespread applications of this bio-ceramic is its weak mechanical properties [26].

### Electrophoretic deposition

Due to the coating's adherence to the substrate, the EPD of biocompatible materials on the metallic substrate is a step in improving dental implants [27]. EPD creates uniformly thick deposits on substrates, consistent with Khora *et al*. [28]. In this study, Cp Ti was coated with HA and CaCO_3_ to demonstrate that electrophoretic deposition processes yield substrates with constant thickness [29].

### Microscopical findings

The surface of coated samples revealed a reasonably uniform distribution of particles, contradicting Chibar *et al*. [30]. Moreover, the micro-topography was beneficial in promoting osseointegration by improving osteoblast adhesion creation of bone on implant surface attachment.

### 2 weeks after implantation

In coated screws, the mixture of HA and CaCO_3_ was released and diffused to reach the osteoblast to stimulate the proliferation and differentiation of osteoplastic cells and inhibit the activity and differentiation of osteoclast cells, resulting in increased matrix deposition and new bone formation [31]. In combination, HA released calcium and phosphate ions that caused (bone-like) apatite to precipitate on the surface of the implant. This bone-like apatite can subsequently trigger cellular differentiation and bone formation as a result (apatite plus osteoblast cells), which results in osseointegration [32].

### 6 weeks after implantation

There was a high amount of bone formation due to the higher bond strength at the implant-bone interface [33].

### Histomorphometric analysis

A characteristic test assesses the histomorphometric properties of the implant-tissue surface [34]. This technique has a high degree of reproducibility and can be used to determine implant stability before, during, or after implantation [35].

## CONCLUSION

When calcium carbonate and nano-hydroxy apatite were used to coat CP Ti implants, the new bone began to develop at the implant interface after two weeks. In addition, the amount of calcified osteoid tissue exhibiting osteoblastic and osteoclastic activity increased six weeks after coated screws were implanted.

## Data Availability

The data used to support the findings of this study are available from the corresponding author upon request.
